# Supplementary figures and images for: Can ephapticity contribute to brain complexity?
Source: PLoS One. 2024 Dec 5;19(12):e0310640. doi: 10.1371/journal.pone.0310640 (PMC11620465; doi:10.1371/journal.pone.0310640)

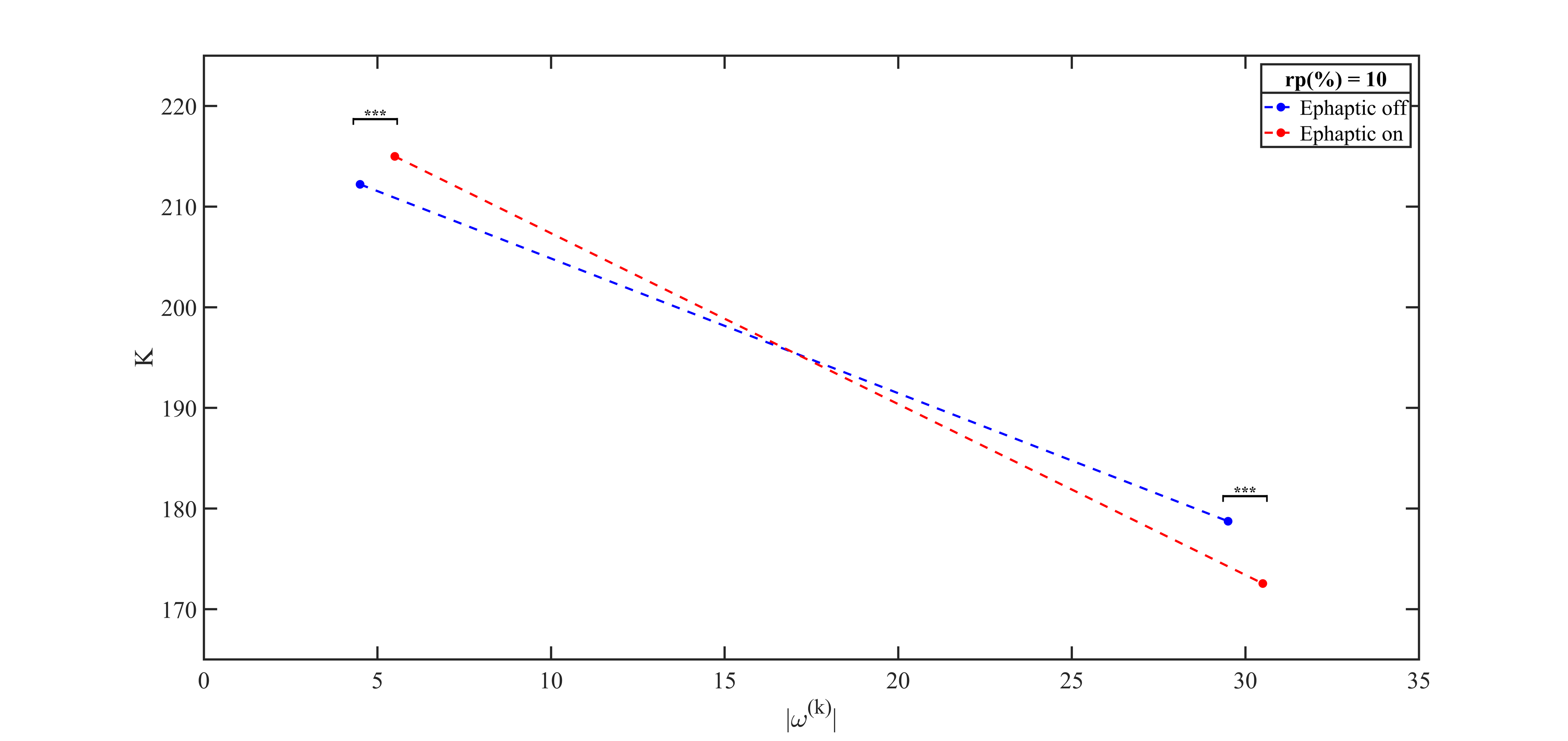

Supplement: S1 Fig — The figure show the complexity for synaptic intensities of |ω(k)| = 5 and |ω(k)| = 30, comparing the scenarios with ephaptic coupling off (blue) and on (red). Notably, the complexity results are analogous to those observed in Fig 3(b) of the main text. These results indicate that increasing synaptic interaction leads to an inversion in complexity values when ephaptic coupling is on versus off. The similarity between the results of purely excitatory and excitatory-inhibitory networks suggests that complexity may exhibit a universal characteristic. This implies that ephaptic coupling enhances communication efficiency in a physiological scenario, regardless of the network’s specific characteristics, connectivity, topology, and scale. (TIF) [file pone.0310640.s001.tif]

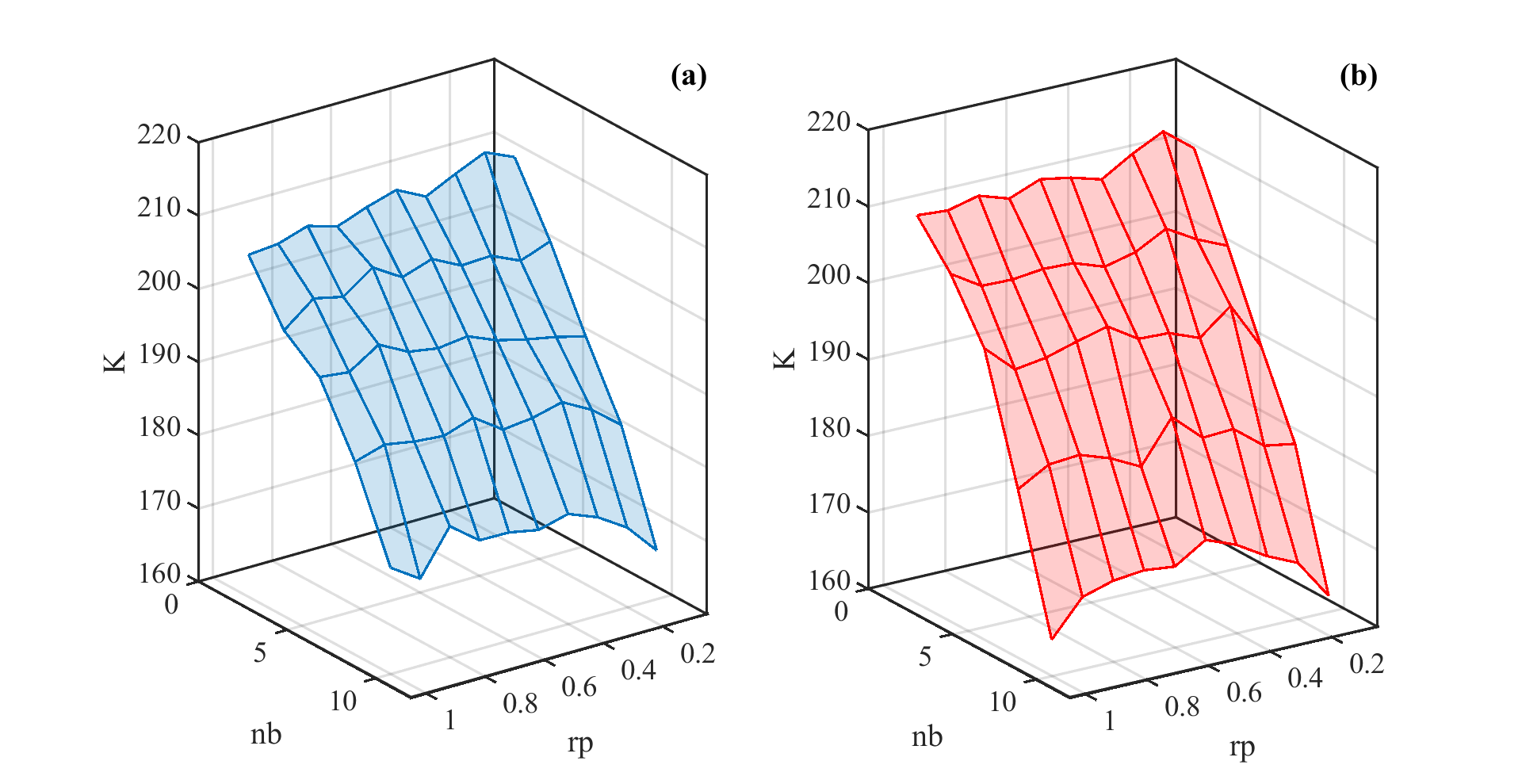

Supplement: S2 Fig — (a) Complexity (K) for different neighbors number (nb) and different rewiring probabilities (rp) values, to networks ephaptic-off small-world. Note that, to values of rp ≈ 10%, and nb = 4, the small-world features promote highest complexity. Otherwise, to nb = 20 the highest values of complexity are presented in random networks structure. (b) Complexity (K) for different neighbors number and different rp values, to networks ephaptic-on. Observe that the complexity values is highest than S1(a) Fig in low nb values. However, the increase of nb promotes an accentuated decrease in comparison with (a). Therefore, the small-world prevalence occurs in minors nb. (TIF) [file pone.0310640.s002.tif]

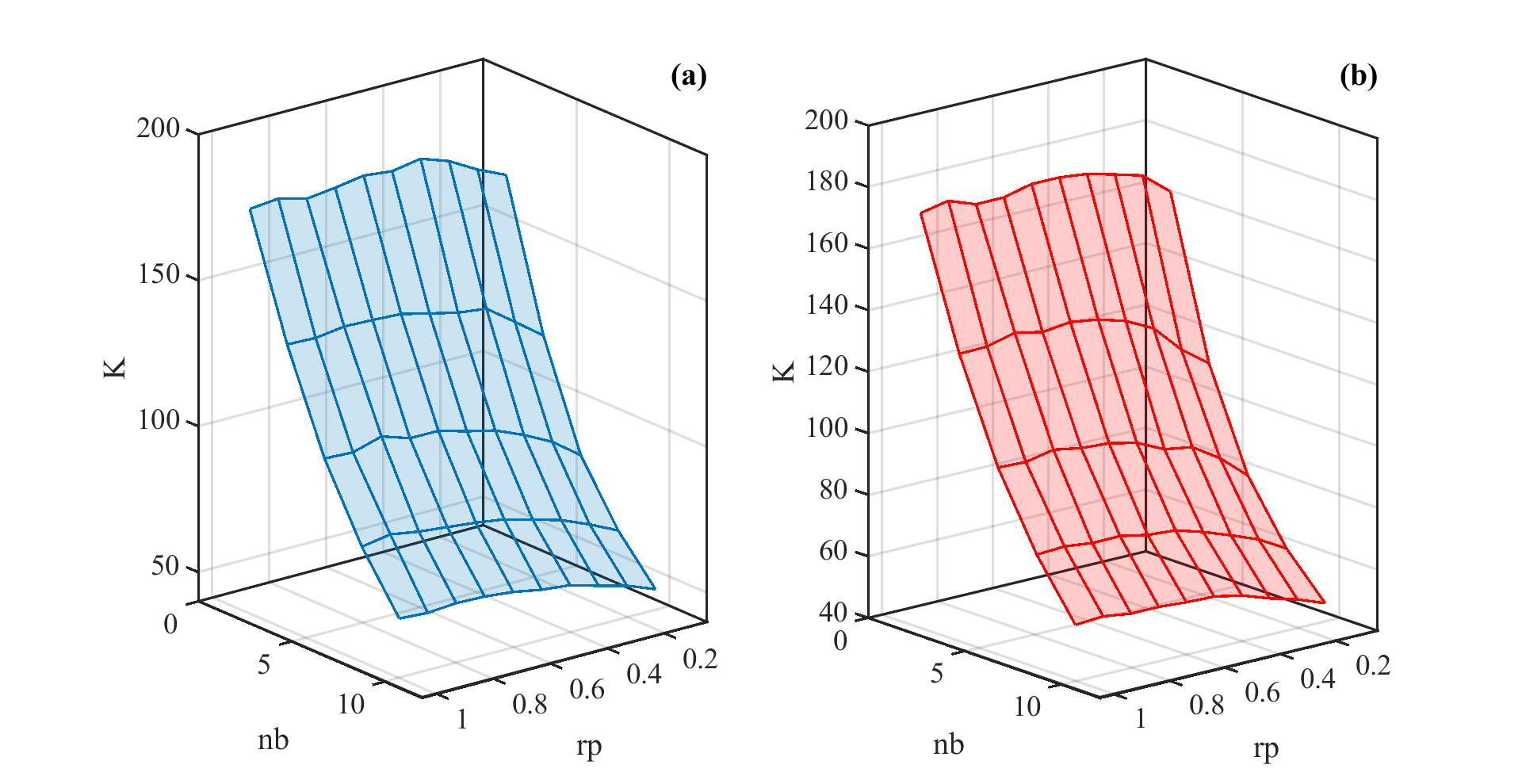

Supplement: S3 Fig — (a) Complexity (K) for different neighbors number (nb) and different rewiring probabilities (rp) values, to networks ephaptic-off small-world. The complexity to strong synapses is lower in comparison with S2(a) Fig, as shows by Figs 3(b) and 4(b) Complexity (K) for for different neighbors number (nb) and different rewiring probabilities (rp) values, to networks ephaptic-on. The values of complexity to combined networks is lower in comparison with (a). This results are in line with the Figs 3(b) and 4. (TIF) [file pone.0310640.s003.tif]

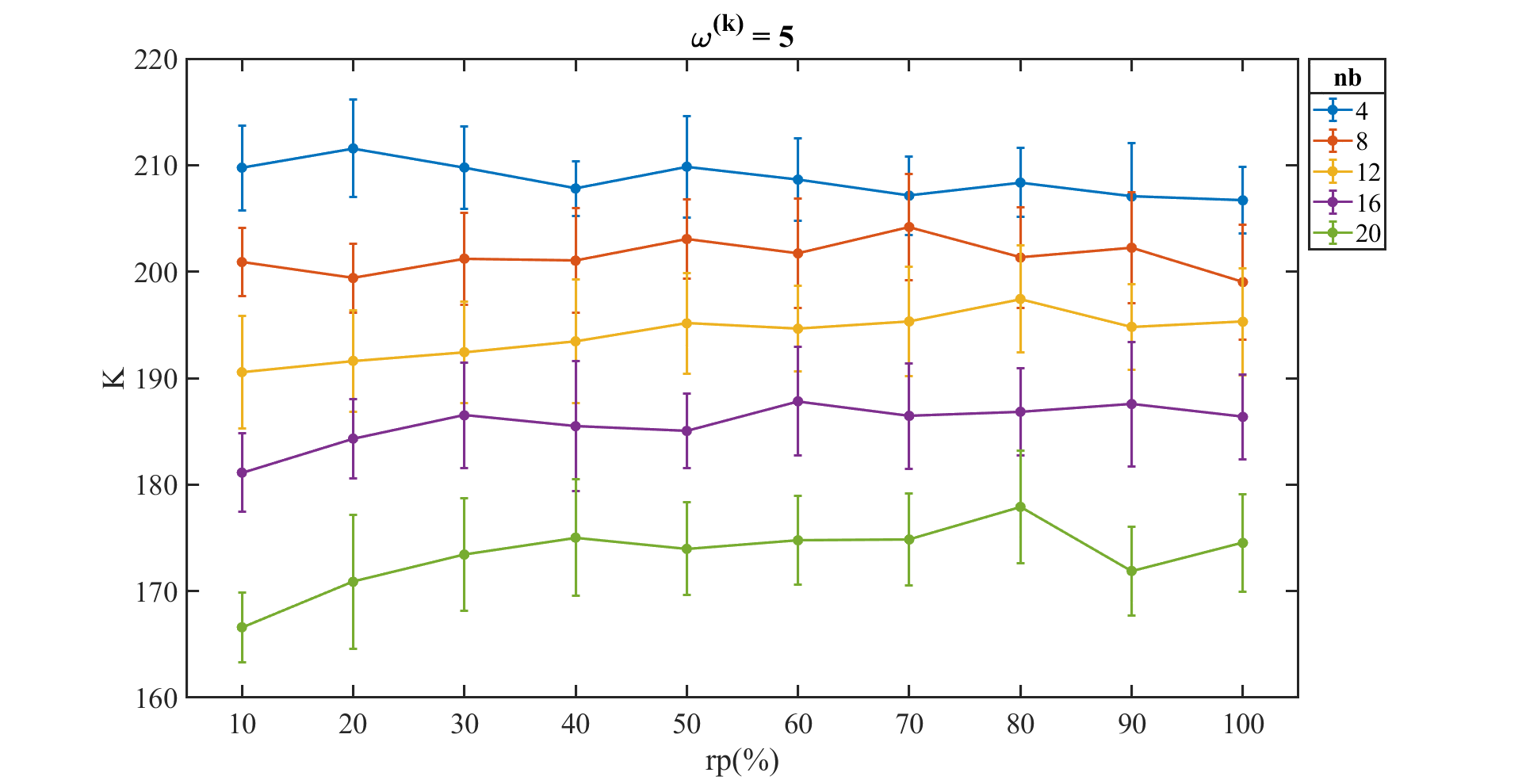

Supplement: S4 Fig — (TIF) [file pone.0310640.s004.tif]

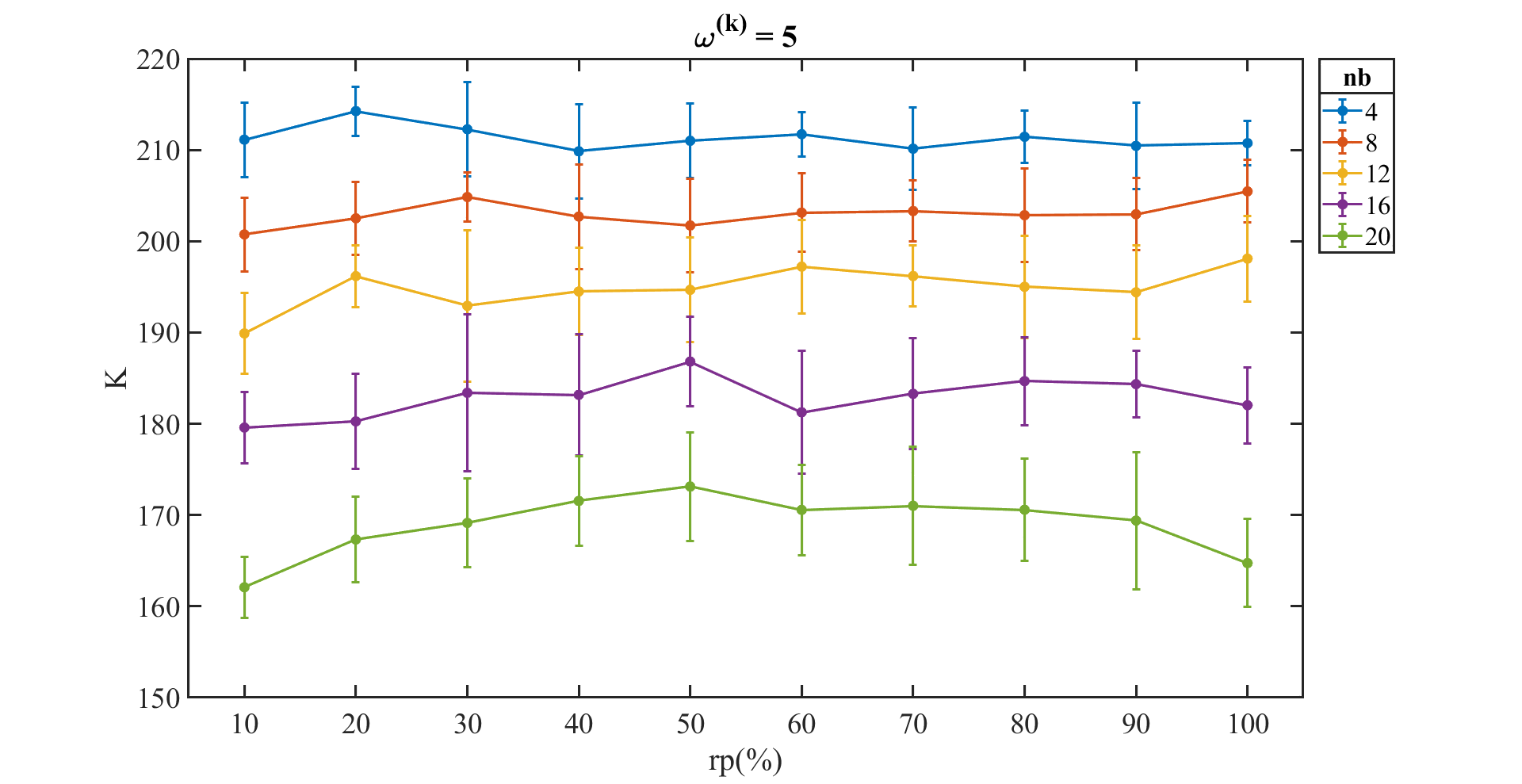

Supplement: S5 Fig — (TIF) [file pone.0310640.s005.tif]

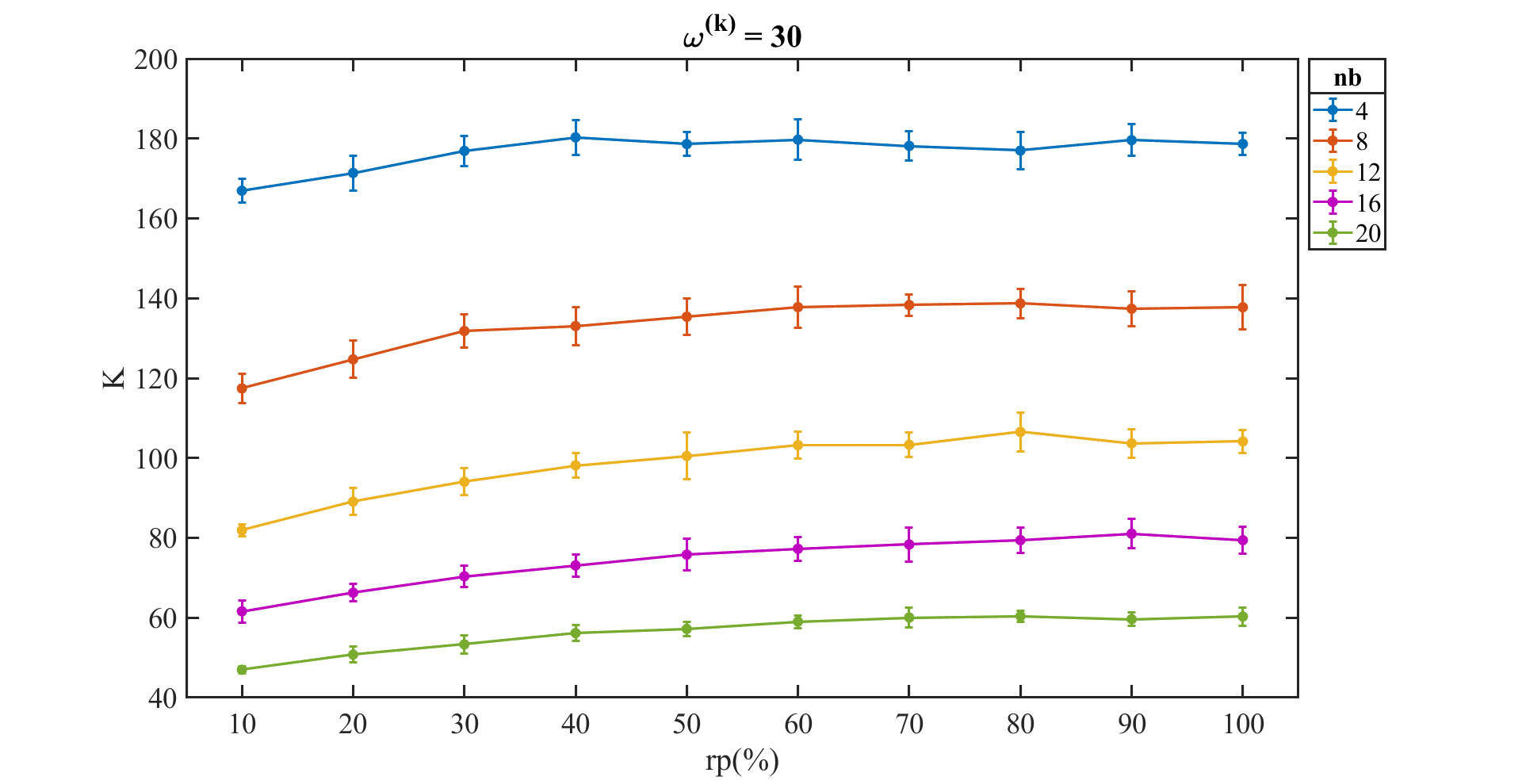

Supplement: S6 Fig — (TIF) [file pone.0310640.s006.tif]

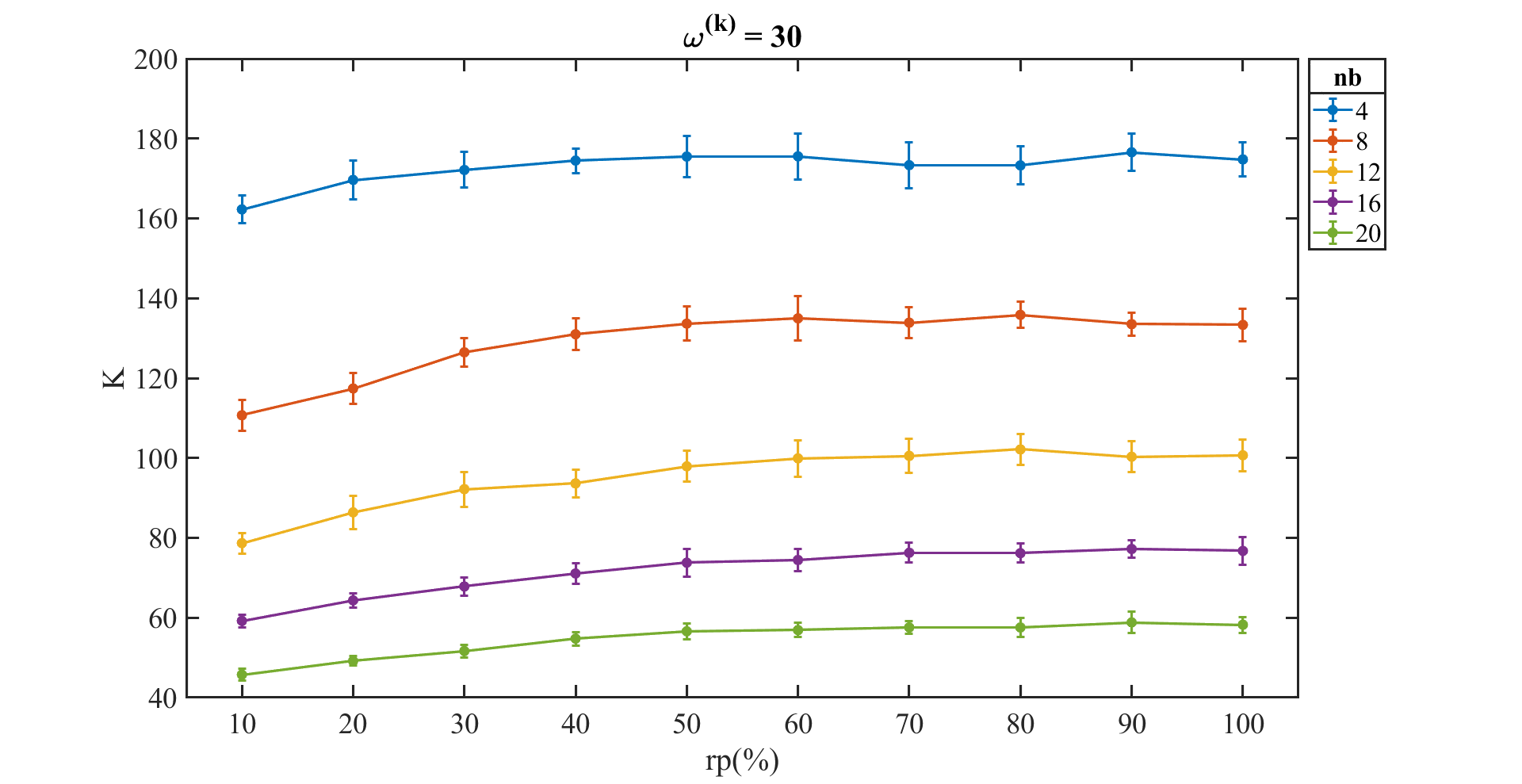

Supplement: S7 Fig — (TIF) [file pone.0310640.s007.tif]
